# Supplementary material for: Oxygen Adsorption Induced Superconductivity in Ultrathin FeTe Film on SrTiO3(001)
Source: Materials (Basel). 2021 Aug 15;14(16):4584. doi: 10.3390/ma14164584 (PMC8398274; doi:10.3390/ma14164584)
Supplement: Supplementary file 1 [file materials-14-04584-s001.zip › materials-1328290-supplementary.pdf]

materials

MDPI

Article

Oxygen Adsorption Induced Superconductivity in Ultrathin FeTe Film on $SrTiO_3(001)$

Wei Ren [1], Hao Ru [2], Kun Peng [3], Huifang Li [3], Shuai Lu [3], Aixi Chen [3], Pengdong Wang [3], Xinwei Fang [3], Zhiyun Li [3], Rong Huang [3], Li Wang [3,*], Yihua Wang [2,4,*] and Fangsen Li [1,3,*]

1 School of Nano-Tech and Nano-Bionics, University of Science and Technology of China, Hefei 230026, China; wren2019@sinano.ac.cn
2 Department of Physics and State Key Laboratory of Surface Physics, Fudan University, Shanghai 200438, China; hru16@fudan.edu.cn
3 Vacuum Interconnected Nanotech Workstation (Nano-X), Suzhou Institute of Nano-Tech and Nano-Bionics (SINANO), Chinese Academy of Sciences (CAS), Suzhou 215123, China; 18752018528@163.com (K.P.); 51171213005@stu.ecnu.edu.cn (H.L.); slu2018@sinano.ac.cn (S.L.); axchen2017@sinano.ac.cn (A.C.); pdwang2020@sinano.ac.cn (P.W.); xwfang2020@sinano.ac.cn (X.F.); zhiyunli2018@sinano.ac.cn (Z.L.); rhuang2009@sinano.ac.cn (R.H.)
4 Shanghai Research Center for Quantum Sciences, Shanghai 201315, China
* Correspondence: lwang2017@sinano.ac.cn (L.W.); wangyhv@fudan.edu.cn (Y.W.); fsli2015@sinano.ac.cn (F.L.)

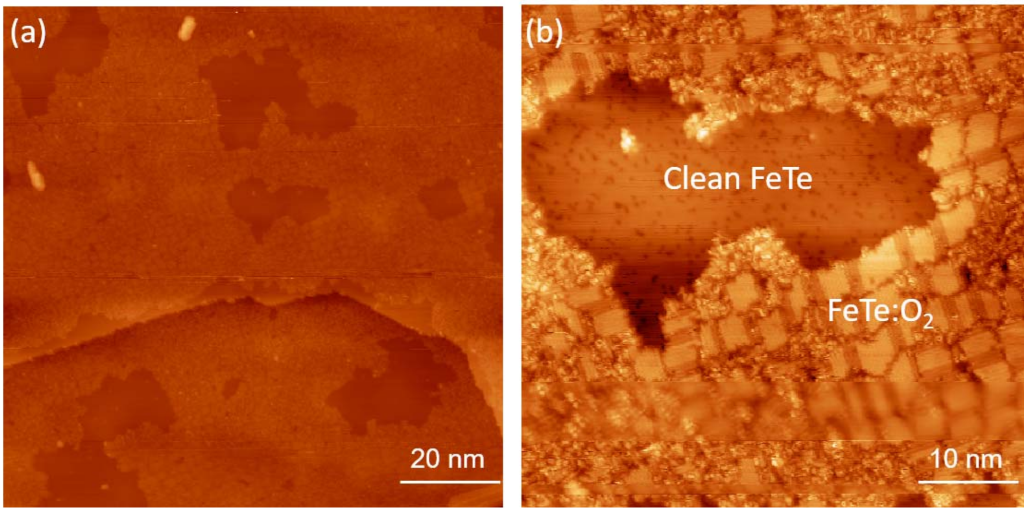

Figure S1. (color online) (**a**) Surface topography (100 nm × 100 nm) of the 10UC FeTe films after exposure to O_2 under 1.6×10^{-4} mbar for 4 hours at room temperature. Scanning condition: V_s=3 V, I_t= 100 pA. (**b**) Enlarged view (50 nm × 50 nm) of surface topography, showing clean FeTe surface with O_2 overlayer. Scanning condition: V_s=1 V, I_t= 100 pA.

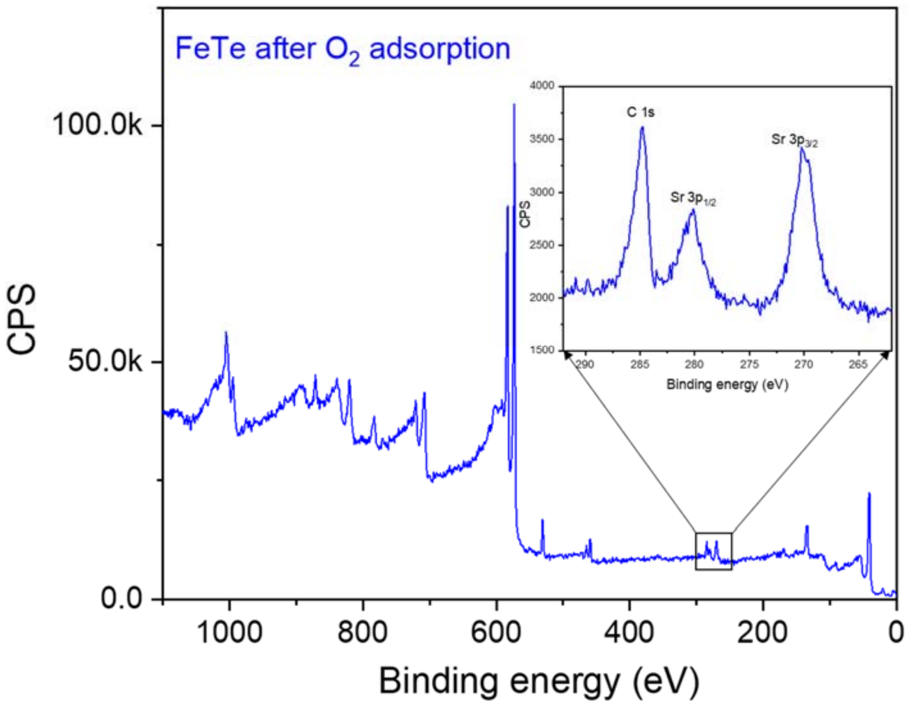

Figure S2. (color online) The XPS survey scan on 10 UC FeTe after O_2 adsorption, showing no other impurities can be observed after O_2 inlet.

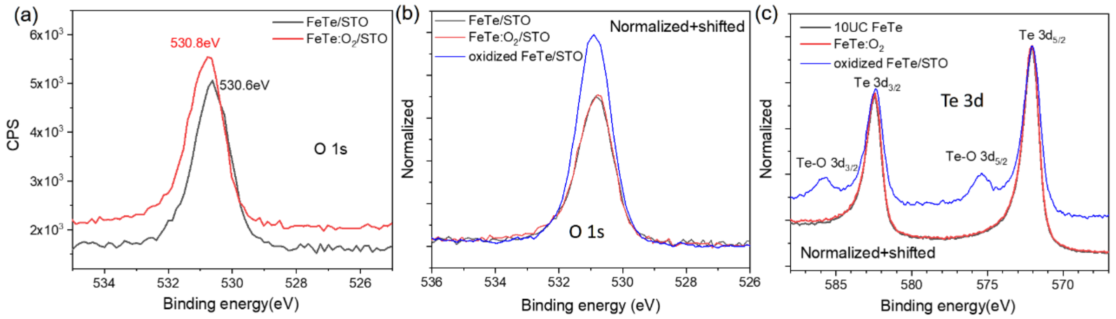

Figure S3. (color online) (**a**)The XPS core level spectra of O1s before and after O_2 inlet, showing similar shift as Te 3d and Fe 2p. (**b, c**) The normalized and shifted XPS core level spectra of O 1s (**b**) and Te 3d (**c**) on 10 UC FeTe before and after O_2 adsorption, showing the shapes of O 1s or Te 3d are nearly overlapped. We also added the XPS spectra on the oxidized FeTe/STO when exposure to O_2 partial pressure of 1.5×10^{-1} mbar for 20 mins.
